# Treatment with Dexamethasone and Monophosphoryl Lipid A Removes Disease-Associated Transcriptional Signatures in Monocyte-Derived Dendritic Cells from Rheumatoid Arthritis Patients and Confers Tolerogenic Features

**DOI:** 10.3389/fimmu.2016.00458

**Published:** 2016-10-25

**Authors:** Paulina A. García-González, Katina Schinnerling, Alejandro Sepúlveda-Gutiérrez, Jaxaira Maggi, Lorena Hoyos, Rodrigo A. Morales, Ahmed M. Mehdi, Hendrik J. Nel, Lilian Soto, Bárbara Pesce, María Carmen Molina, Miguel Cuchacovich, Milton L. Larrondo, Óscar Neira, Diego Francisco Catalán, Catharien M. Hilkens, Ranjeny Thomas, Ricardo A. Verdugo, Juan C. Aguillón

**Affiliations:** ^1^Programa Disciplinario de Inmunología, Facultad de Medicina, Instituto de Ciencias Biomédicas (ICBM), Universidad de Chile, Santiago, Chile; ^2^Millennium Institute on Immunology and Immunotherapy, Santiago, Chile; ^3^Programa de Genética Humana, ICBM, Facultad de Medicina, Universidad de Chile, Santiago, Chile; ^4^Translational Research Institute, University of Queensland Diamantina Institute, Woolloongabba, QLD, Australia; ^5^Unidad de Dolor, Hospital Clínico de la Universidad de Chile, Santiago, Chile; ^6^Departamento de Medicina, Hospital Clínico de la Universidad de Chile, Santiago, Chile; ^7^Banco de Sangre, Hospital Clínico de la Universidad de Chile, Santiago, Chile; ^8^Sección de Reumatología, Hospital del Salvador, Santiago, Chile; ^9^Musculoskeletal Research Group, Faculty of Medical Sciences, Institute of Cellular Medicine, Newcastle University, Newcastle upon Tyne, UK

**Keywords:** rheumatoid arthritis, dendritic cells, tolerance, cell-based therapy, transcriptome

## Abstract

Tolerogenic dendritic cells (TolDCs) are promising tools for therapy of autoimmune diseases, such as rheumatoid arthritis (RA). Here, we characterize monocyte-derived TolDCs from RA patients modulated with dexamethasone and activated with monophosphoryl lipid A (MPLA), referred to as MPLA-tDCs, in terms of gene expression, phenotype, cytokine profile, migratory properties, and T cell-stimulatory capacity in order to explore their suitability for cellular therapy. MPLA-tDCs derived from RA patients displayed an anti-inflammatory profile with reduced expression of co-stimulatory molecules and high IL-10/IL-12 ratio, but were capable of migrating toward the lymphoid chemokines CXCL12 and CCL19. These MPLA-tDCs induced hyporesponsiveness of autologous CD4+ T cells specific for synovial antigens *in vitro*. Global transcriptome analysis confirmed a unique transcriptional profile of MPLA-tDCs and revealed that RA-associated genes, which were upregulated in untreated DCs from RA patients, returned to expression levels of healthy donor-derived DCs after treatment with dexamethasone and MPLA. Thus, monocyte-derived DCs from RA patients have the capacity to develop tolerogenic features at transcriptional as well as at translational level, when modulated with dexamethasone and MPLA, overcoming disease-related effects. Furthermore, the ability of MPLA-tDCs to impair T cell responses to synovial antigens validates their potential as cellular treatment for RA.

## Introduction

Rheumatoid arthritis (RA) is a chronic autoimmune disease characterized by the inflammation of synovial tissue, which leads to progressive cartilage and bone destruction. The development of RA is attributed to an uncontrolled activation of auto-reactive CD4+ T cells (1, 2). Dendritic cells (DCs) play a pivotal role in the pathogenesis of RA through presentation of autoantigen peptides on major histocompatibility complex (MHC) molecules (3, 4). The secretion of pro-inflammatory cytokines by DCs further promotes the maturation of bystander DCs and drives the differentiation of T helper type 1 (Th1) and Th17 cells, both involved in the pathology and progression of RA (1, 2).

Current treatment of RA includes the use of immunosuppressive drugs and/or biologic therapies (2, 5). All of them require lifelong treatment and, thus, new therapeutic strategies are needed to suppress inflammation and re-establish self-tolerance without affecting the whole patient’s immune system. In particular, research has focused on the generation of tolerogenic DCs (TolDCs) capable of modulating antigen-specific immune responses (6, 7). TolDCs may induce antigen-specific tolerance through induction of anergy, deletion of auto-reactive T cells (8, 9), generation and expansion of regulatory T cells (Treg) (10, 11), and/or the establishment of an anti-inflammatory environment through secretion of IL-10 and/or transforming growth factor beta (TGF-β) (12).

Different strategies have been described for the *in vitro* generation of TolDCs derived from peripheral blood monocytes, including modulation by IL-10 or TGF-β (8, 13), dexamethasone (14), Bay11-7082 (15), rapamycin (16), aspirin (17) or vitamin D3 (18), short stimulation with lipopolysaccharide (LPS) (19), or its non-toxic analog monophosphoryl lipid A (MPLA) (20). Although TolDC properties may vary according to the applied protocol, TolDC features include reduced expression of co-stimulatory and antigen presentation molecules, low IL-12 production, and suppression of effector T cell responses *in vitro* (21, 22). Application of TolDCs for RA treatment has been successfully tested in animal models (23, 24). Phase I clinical trials using *in vitro* modified autologous TolDCs demonstrated feasibility and safety in patients with type 1 diabetes (25) and RA (15, 26).

Recently, we described a shortened protocol for the differentiation of monocytes from healthy donors into TolDCs, using dexamethasone for immunomodulation, and current good manufacture practice (cGMP)-grade MPLA to trigger toll-like receptor (TLR)-mediated activation, including the upregulation of chemokine receptors that mediate the migration to secondary lymphoid organs (20). These MPLA-tDCs expressed low levels of co-stimulatory molecules and maturation markers, and secreted high levels of IL-10 and low levels of IL-12. In functional analyses, they migrated to lymphoid chemokines and induced lower levels of T cell proliferation and cytokine production than mature DCs (27).

Monocytes from RA patients were demonstrated to exhibit a highly inflammatory profile (28, 29) and studies investigating their capacity to develop into functional TolDCs *in vitro* showed contradictory results (30, 31), suggesting that disease-associated factors might affect TolDC differentiation. To date, there are no studies comparing the transcriptomes of immature, mature, and modulated monocyte-derived DCs (moDCs) from RA patients and healthy subjects.

Therefore, the aim of the present study was to translate our MPLA-tDC protocol to moDCs derived from RA patients, and to characterize them at phenotypic, functional, and transcriptional level in order to validate their applicability as autologous cellular therapy to restore antigen-specific tolerance in RA.

## Materials and Methods

The minimum information about tolerogenic antigen-presenting cells (MITAP) checklist (32) was followed for the preparation of this manuscript.

### Blood Samples and Synovial Fluid

Twenty-seven leukapheresates from patients with active RA and 28 buffy coats from healthy donors were obtained from Hospital del Salvador and Hospital Clínico de la Universidad de Chile. Demographic characteristics of patients and healthy donors are detailed in Table S1 in Supplementary Material. All RA patients fulfilled ACR criteria for RA diagnosis and received treatment as described in Table S1 in Supplementary Material. Subjects signed an informed written consent according to the Declaration of Helsinki and all procedures were approved by the Ethics Committees of the Facultad de Medicina and Hospital Clínico from Universidad de Chile, and Hospital del Salvador.

Synovial fluid (SF) was collected through arthrocentesis of inflamed knees of one RA patient. Removal of cells from SF was done by centrifugation at 1800 rpm for 5 min. The acellular fraction was treated with hyaluronidase (100 U/ml) for 60 min at 37°C to reduce viscosity and centrifuged at 1800 rpm for 10 min before passing through a 0.2-μm filter. Protein concentration was quantified using the BCA method (Sigma-Aldrich, MO, USA) at A_562_ (Table S1 in Supplementary Material).

### Generation of Monocyte-Derived DCs

Monocytes were isolated by negative selection using RosetteSep Human Monocyte enrichment cocktail (Stemcell Technologies, Vancouver, BC, Canada) according to manufacturer’s instructions. moDCs were generated as previously described (20) in AIM-V medium (Gibco BLR, Grand Island, NE, USA), supplemented with 500 U/ml of recombinant human GM-CSF and IL-4 (eBioscience, San Diego, CA, USA) within 5 days. At days 3 and 4, cells were modulated with 1 μM dexamethasone (tDCs) (Sigma-Aldrich, St. Louis, MO, USA) and activated with 1 μg/ml cGMP-grade MPLA (MPLA-tDCs) (Avanti, Alabaster, AL, USA). Untreated/immature DCs (iDCs) and MPLA-matured DCs (mDCs) were used as controls.

### Flow Cytometry

Antibodies used for analysis were anti-human CD11c APC, CD80 FITC, CD83 FITC, HLA-DR FITC, CD40 PE, CD86 PE, TLR-2 PE, CXCR4 PE, CCR7 PE, CD4 PECy7, and IFN-γ APC (all from eBioscience). Cells were resuspended in PBS supplemented with fetal bovine serum (FBS) (HyClone Thermo Scientific, Logan, UT, USA), stained with specific antibodies, fixed with IC fixation buffer (eBioscience) and resuspended in FACSFlow buffer (Becton Dickinson, San Diego, CA, USA) for subsequent analysis. Data were acquired on a FACSAria III with FACSDiva v6.1.3 software (both Becton Dickinson) and analyzed by FlowJo software (Treestar, USA).

### Cytokine Production

1 × 10^5^ DCs were incubated for 24 h with or without CD40L-transfected irradiated NIH3T3 cells at 1:1 ratio in AIM-V medium in 96-well U bottom plates (BRAND, Wertheim, Germany). Supernatants of co-cultures with NIH3T3 cells or T cells were recovered and stored at −80°C until quantification of IL-10, IL-12p70, IL-23, TNF-α, TGF-β1(active), and IL-17A by ELISA (eBioscience).

### Chemotaxis Assay

Migration was assessed *in vitro* using a transwell system (24-well, pore size 5 μm polycarbonate inserts; Corning Costar, USA). 1.5 × 10^5^ DCs from RA patients were seeded in the upper chamber and AIM-V medium alone or supplemented with 250 ng/ml of SDF-1α/CXCL12 or MIP-3β/CCL19 (PeproTech, Rocky Hill, CT, USA) was added to the lower chamber. After 4-h incubation at 37°C and 5% CO_2_, DCs in the lower chamber were counted by flow cytometry. DCs migration is expressed as “migration index” (migration toward chemokines/migration toward medium).

### Assessment of CD4+ T Cell-Stimulatory Capacity of DCs

CD4+ T cells were isolated by negative selection using RosetteSep Human T cell enrichment cocktail (Stemcell Technologies, Vancouver, BC, Canada) and labeled with carboxyfluorescein diacetate succinimidyl ester (CFSE).

For allogeneic assays, DCs from RA patients were co-cultured with CD4+ T cells from healthy donors. For assessment of antigen-specific CD4+ T cell activation, DCs from RA patients, loaded with 1 μg/ml tuberculin purified protein derivative (PPD; Staten Serum Institute, Copenhagen, Denmark) or 200 μg/ml SF proteins 4 h prior to activation with MPLA, were co-cultured with autologous CD4+ T cells at a DC:T cell ratio of 1:2 in RPMI medium (HyClone Thermo Scientific) with 10% FBS in 96-well U bottom plates for 6 days (20). CD4+ T cells alone, or stimulated with anti-human CD3 (eBioscience) were used as negative and positive controls, respectively. Supernatants were collected to assess cytokine secretion. For intracellular IFN-γ detection, 50 ng/ml phorbol-12-myriastate-13-acetate (PMA, Sigma-Aldrich), 1 μg/ml ionomycin (Sigma-Aldrich), and 1 μg/ml brefeldin-A (eBioscience) were added for the last 5 h of culture. Proliferation and IFN-γ production of CD4+ T cells were analyzed by flow cytometry.

### RNA Isolation and Microarray Analysis

Total RNA was isolated from 5 × 10^5^ moDCs using RNeasy Mini Kit (Qiagen, Hilden, Germany) according to manufacturer’s instructions. Yield and quality of RNA samples were evaluated with NanoDrop 1000 spectrophotometer (Thermo Scientific, Waltham, MA, USA) and RNA Integrity (RIN score) was analyzed with Agilent 2100 Bioanalyzer (Agilent Technologies, Santa Clara, CA, USA) or LabChip GX/GX II (Caliper LifeSciences, Hopkinton, MA USA). A total of 76 samples, corresponding to DCs derived from 9 RA patients and 10 healthy donors under 4 experimental conditions were considered for microarray analysis (Figure S1 in Supplementary Material). All RNA samples used for microarrays showed A260/A280 values between 1.8 and 2.2, and RIN scores >7. RNA samples were reverse transcribed, amplified, and labeled using a Illumina^®^ TotalPrepTM RNA Amplification Kit, and cRNA was hybridized onto Illumina Human HT-12 v4 BeadChips (Illumina, San Diego, CA, USA), covering the whole human genome. Expression data were extracted with GenomeStudio Project Software from Illumina.

### Data Exploration and Statistical Methods

Friedman repeated measures test (differences between culture conditions) or Kruskal–Wallis test (differences between RA patients and healthy donors) and Dunn’s post test were used for comparisons. Wilcoxon signed-rank test was used to compare migration of different DC populations. Analyses were performed using Prism 5.01 software (Graphpad, San Diego, CA, USA). *P*-values ≤0.05 were considered significant.

Microarray data were log-transformed and normalized using the preprocess Core package v1.28.0 from Bioconductor. Principal component analysis (PCA) was performed using the PCA function of mixOmics package v5.0-3 (33) of R software (34), in order to assess differences in differentiation protocols and to detect outliers that might affect downstream analyses. Differentially expressed (DE) genes in modulated DCs compared to unstimulated DCs (iDCs) were identified with the Maanova package v1.36.0 *T*-test for gene pairwise comparisons (35). A false discovery rate (FDR) of 0.05 or lower was used as cut-off value. K-means clustering of DE genes between MPLA-tDCS and iDCs was performed using the cluster package. Detailed analyses are described in Figure S1 in Supplementary Material.

## Results

### MPLA-tDCs from RA Patients Exhibit a Tolerogenic Phenotype

**Figure 1:**
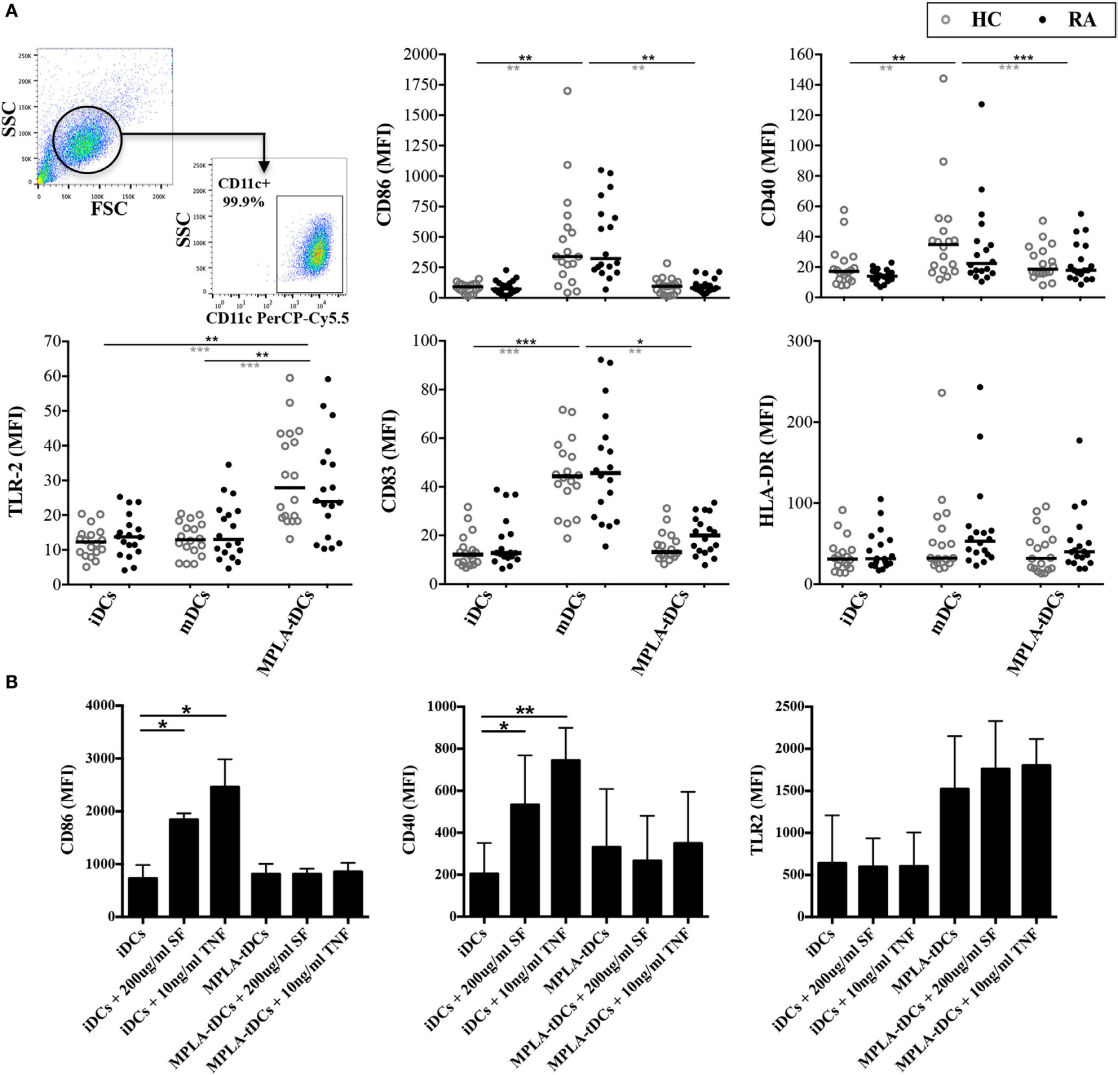
**MPLA-tDCs from rheumatoid arthritis patients and healthy controls display a tolerogenic phenotype that remains unaffected under pro-inflammatory conditions**. **(A)** Monocytes from patients with rheumatoid arthritis (RA) (*black closed symbols*) and from healthy control (HC) subjects (*gray open symbols*) were differentiated into DCs in the presence of GM-CSF and IL-4 within 5 days. For tolerization, DCs were conditioned with dexamethasone (Dex) during 48 h and were additionally activated with MPLA for the last 24 h of culture (MPLA-tDCs). Surface expression levels of CD86, CD40, CD83, HLA-DR, and TLR2 were assessed by flow cytometry. Dot plots show all data points and median values are indicated as a line. **(B)** MPLA-tDCs from RA patients were incubated for 72 h in the presence of synovial fluid (200 μg/ml) or TNFα (10 ng/ml), and the expression of phenotypic markers was analyzed. Untreated iDCs were used as controls. Statistical differences were calculated using either Kruskal–Wallis test (comparison between HC and RA) or Friedman test (comparison between culture conditions) and Dunn’s multiple comparison was used as post test (*P < 0.05; **P < 0.01; ***P < 0.001).

The phenotype of different DCs subsets, generated from monocytes of RA patients and healthy subjects was analyzed by flow cytometry. All DC populations contained >95% CD11c+ cells and exhibited high CD1a and low CD14 expression, while their viability ranged from 70 to 90% and did not differ between RA patients and healthy donors (data not shown). In comparison to mDCs, dexamethasone-treated and MPLA-activated DCs (MPLA-tDCs) showed reduced expression of CD86 and CD83, similar to iDCs (Figure 1A). Expression of CD40 and CD80 was also reduced in MPLA-tDCs compared to mDCs (Figure 1A and data not shown). In accordance with the previously described elevated expression of TLR2 on TolDC generated with dexamethasone, vitamin D3, and MPLA (31), we observed that TLR2 expression was significantly increased on MPLA-tDCs as compared to iDCs and mDCs (Figure 1A). No significant differences were found concerning the phenotype of DC subsets between healthy subjects and RA patients (Figure 1A). Furthermore, exposure of RA-derived MPLA-tDCs to inflammatory stimuli, such as SF or TNF, did not affect the tolerogenic phenotype of these cells (Figure 1B).

### MPLA-tDCs from RA Patients Show an Anti-Inflammatory Cytokine Profile

**Figure 2:**
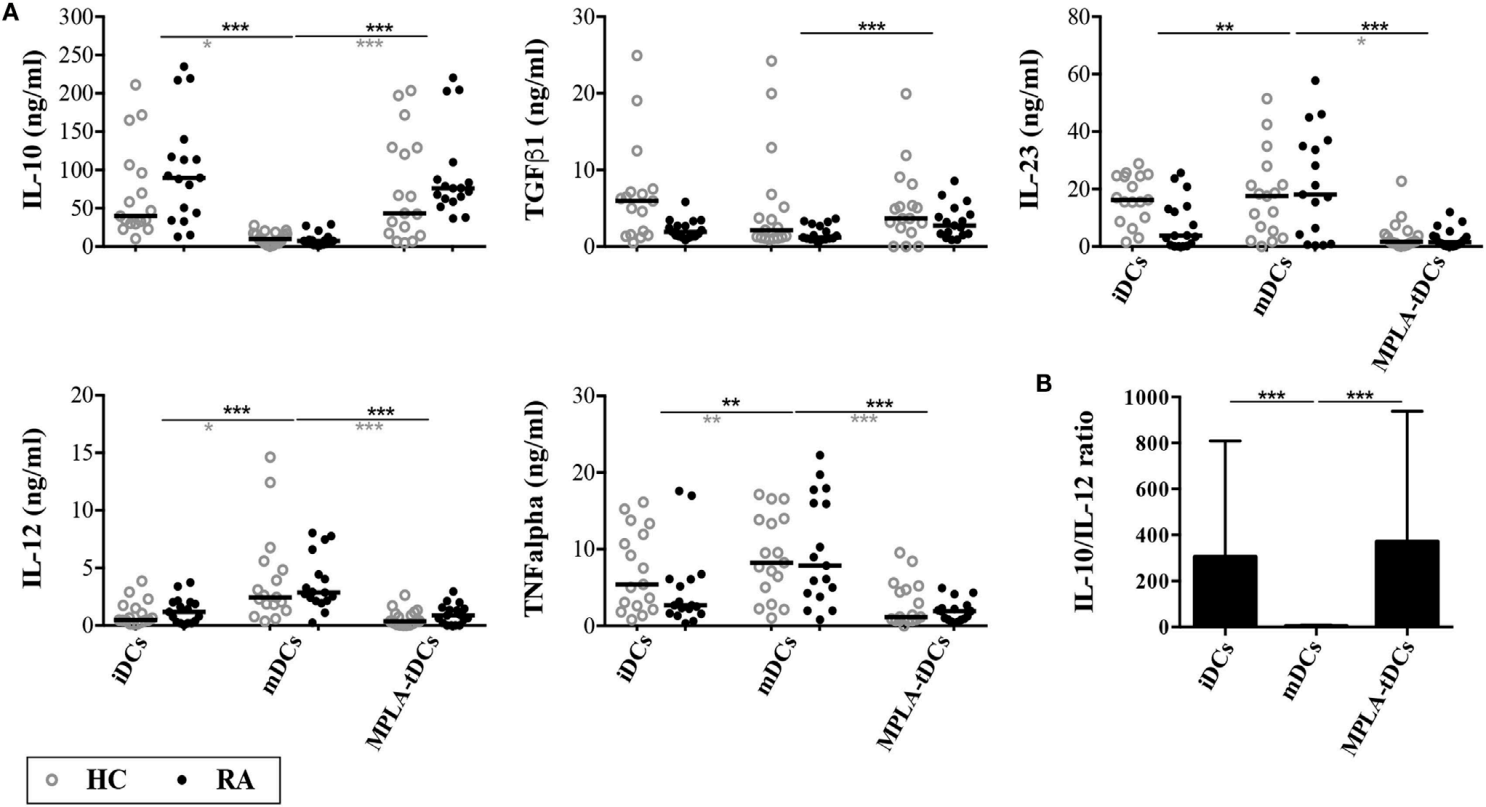
**MPLA-tDCs from rheumatoid arthritis patients exhibit an anti-inflammatory cytokine profile**. Monocyte-derived DC (moDC) subsets from rheumatoid arthritis (RA) patients (*black closed symbols*) and healthy control subjects (HC; *gray open symbols*) were cultured with CD40L-transfected NIH3T3 cells at 1:1 ratio for 24 h. **(A)** Concentrations of IL-10, IL-12, IL-23, TNF-α, and active TGF-β1 were determined in culture supernatants of moDCs by ELISA. **(B)** Bar chart with values of IL-10/IL-12 ratio of different moDC subsets from RA patients. Dot plots show all data points and horizontal lines indicate median values. Statistical differences were calculated using Kruskal–Wallis test (comparison of HC and RA) or Friedman test (comparison of DC types) and Dunn’s multiple comparison post test (*P < 0.05; **P < 0.01; ***P < 0.001).

Consistent with our previous findings in healthy subjects (20), we found that MPLA-tDCs from RA patients produced 10-fold more IL-10 and 5-fold less IL-12p70 than mDCs in response to CD40 ligation (Figure 2). Although IL-10 levels tended to be higher in DCs from RA patients than in those from healthy subjects, these differences were not significant. MPLA-tDCs secreted significantly lower levels of TNF-α and IL-23 and higher levels of TGF-β1 than mDCs. The cytokine profiles of MPLA-tDCs and iDCs derived from RA patients were similar to those from healthy donors (Figure 2). Interestingly, the rate of IL-10/IL-12 in MPLA-tDCs from RA patients was significantly higher than that observed in mDCs (Figure 2B). Mirroring their behavior toward SF and TNFα exposure, MPLA-tDCs maintained their low expression of CD86, CD80, CD40, and CD83, and the high expression of TLR2 upon CD40 engagement (data not shown).

### MPLA-tDCs from RA Patients Migrate toward Secondary Lymphoid Tissue Chemokines

**Figure 3:**
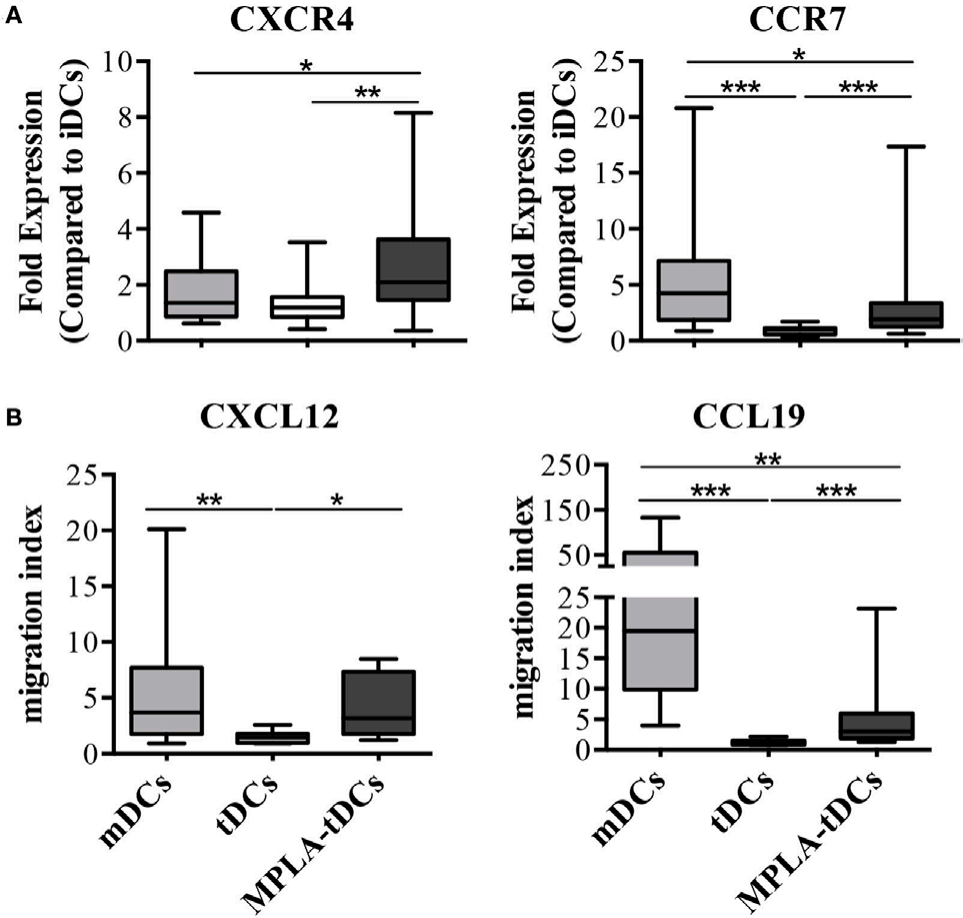
**MPLA-tDCs from rheumatoid arthritis patients display the capacity to migrate toward CCR7 and CXCR4 ligands**. After 5 days of culture, the migratory capacity of MPLA-tDC (*dark gray*), tDCs (*white*) and mDCs (*light gray*) from rheumatoid arthritis (RA) patients was studied *in vitro*. **(A)** The percentage of CD11c+ DCs expressing CXCR4 or CCR7 was determined by flow cytometry (*n* = 15). Fold expression changes on receptor expression on mDCs, tDCs and MPLA-tDCs compared to untreated iDCs are shown. **(B)** Transwell assays were used to assess migration of DCs in response to 250 ng/ml of CXCL12 or CCL19 (*n* = 12). Migration index indicates the quotient of cells that migrated toward a specific chemokine divided by cells that migrated toward medium alone. Box plots show median, 25- and 75%-quartile, minimum and maximum values, respectively. Wilcoxon signed-rank test was used to evaluate statistical differences between MPLA-tDCs, tDCs, and mDCs (*P < 0.05; **P < 0.01; ***P < 0.001).

We investigated the expression of lymph node homing chemokine receptors on DCs from RA patients and their migratory response toward the corresponding chemokines in a transwell system. Expression of CXCR4 and CCR7 was 2.5 fold higher on MPLA-tDCs than in iDCs and significantly enhanced when compared to tDCs (Figure 3A). MPLA-tDCs and mDCs also showed high migration indexes toward the CXCR4 and CCR7 ligands, CXCL12 and CCL19, respectively, exhibiting a higher migratory ability towards lymphoid chemokines than tDCs (Figure 3B).

### MPLA-tDCs from RA Patients Modulate Allogeneic and Autologous T Cell Responses

**Figure 4:**
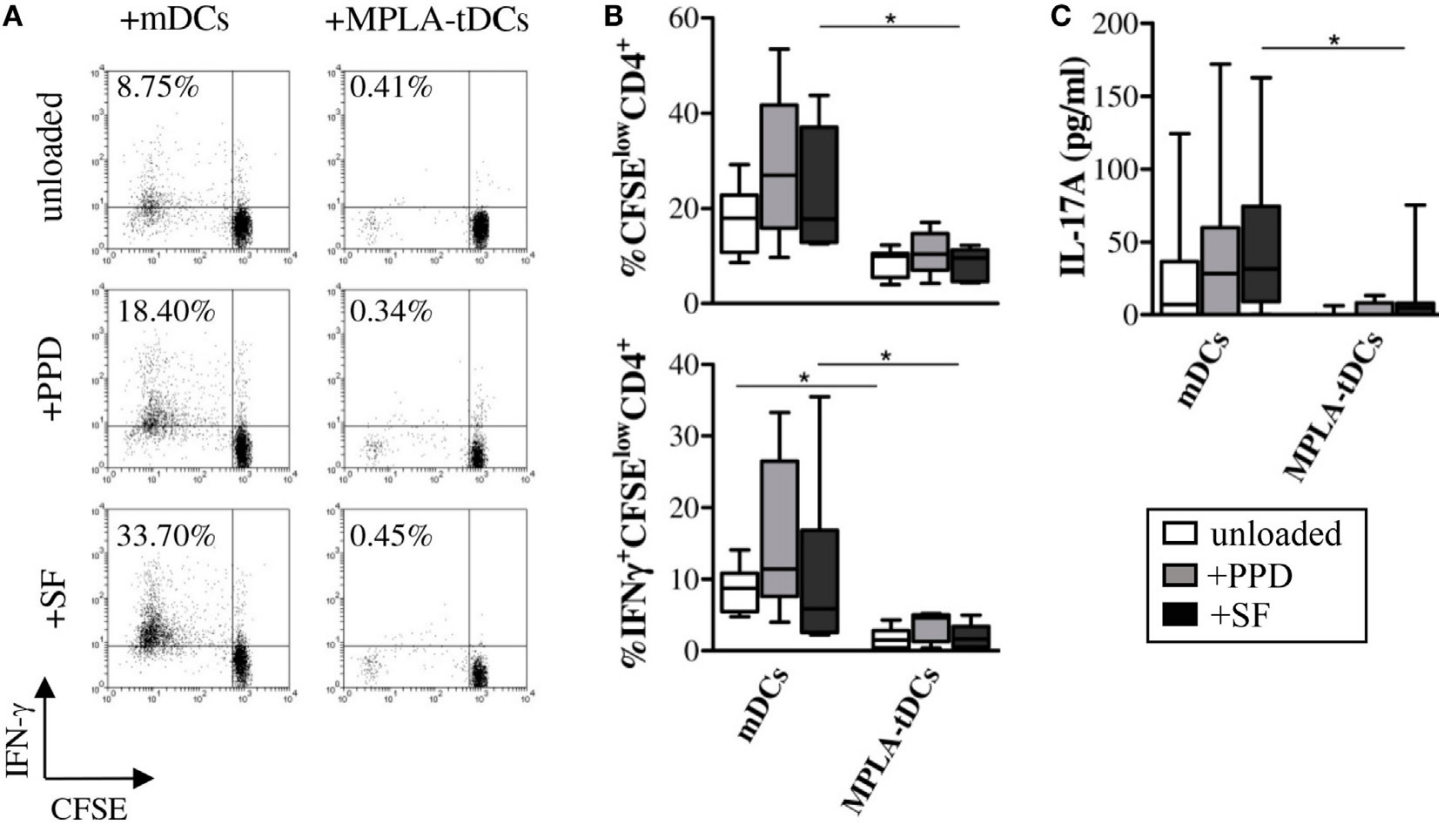
**MPLA-tDCs modulate CD4+ T cell responses to synovial antigens**. Monocytes (*n* = 6–9) were differentiated into mDCs and MPLA-tDCs as described in the Section “Materials and Methods.” At day 4 of DC generation, 4 h prior to stimulation with MPLA, mDCs and MPLA-tDCs were loaded with 1 μg/ml tuberculin purified protein derivative (PPD), 200 μg/ml synovial fluid (SF), or left unloaded for further 24 h. For the assessment of their T cell-stimulatory capacity, antigen-loaded or unloaded mDCs and MPLA-tDCs were co-cultured with autologous CFSE-labeled CD4+ T cells in a ratio of 1:2 (DC:T cell) for 6 days. To detect IFN-γ intracellularly, cells were stimulated with 50 ng/ml phorbol-12-myriastate-13-acetate and 1 μg/ml ionomycin in the presence of 1 μg/ml brefeldin-A. Proliferation-associated CFSE dilution and IFN-γ production of CD4+ T cells were analyzed by flow cytometry. **(A)** Representative dot plots and **(B)** graphic representation of the percentage of IFN-γ-producing proliferating (CFSE^low^) CD4+ T cells are shown. **(C)** IL-17A secretion was measured in supernatants of co-cultures by ELISA. **(B,C)**, Box plots show median, 25- and 75%-quartiles and both extreme values (*P < 0.05).

In order to evaluate T cell responses, MPLA-tDCs, mDCs, or iDCs of RA patients were cultured with allogeneic CD4+ T cells from healthy donors in mixed leukocyte reactions. When compared to mDCs, CD4+ T cell proliferative responses (median %CFSE^low^CD4+ T cells in co-cultures with: iDCs = 13.9; mDCs = 65.4; MPLA-tDCs = 22.8; mDCs vs. MPLA-tDCs: *P* = 0.001) and IFN-γ production (median %IFN-γ + CFSE^low^CD4+ T cells in co-cultures with: iDCs = 3.4; mDCs = 29.7; MPLA-tDCs = 3.0; mDCs vs. MPLA-tDCs: *P* = 0.005) to MPLA-tDCs were significantly reduced (Figure 4).

To investigate the capacity of MPLA-tDCs to present antigens and thereby activate antigen-specific memory T cells, DCs from RA patients were loaded with PPD or SF and co-cultured with autologous CD4+ T cells. While mDCs induced potent proliferation and IFN-γ production of CD4+ T cells, MPLA-tDCs stimulated poor proliferation and IFN-γ expression in SF-specific CD4+ T cells (Figures 4A,B). Only mDCs but not MPLA-tDCs induced the secretion of IL-17A by SF-specific CD4+ T cells (Figure 4C). Proliferation, IFN-γ production, and IL-17A secretion of CD4+ T cells in co-culture with PPD-loaded MPLA-tDCs were slightly, but not significantly lower than those in co-culture with PPD-loaded mDCs (Figures 4B,C). Taken together, these results show that MPLA-tDCs obtained from RA patients have an impaired capacity to induce inflammatory CD4+ T cell responses.

### Modulation with Dexamethasone and MPLA Generates a Particular Transcriptional Profile in DCs, Which Overcomes RA-Associated Effects

**Figure 5:**
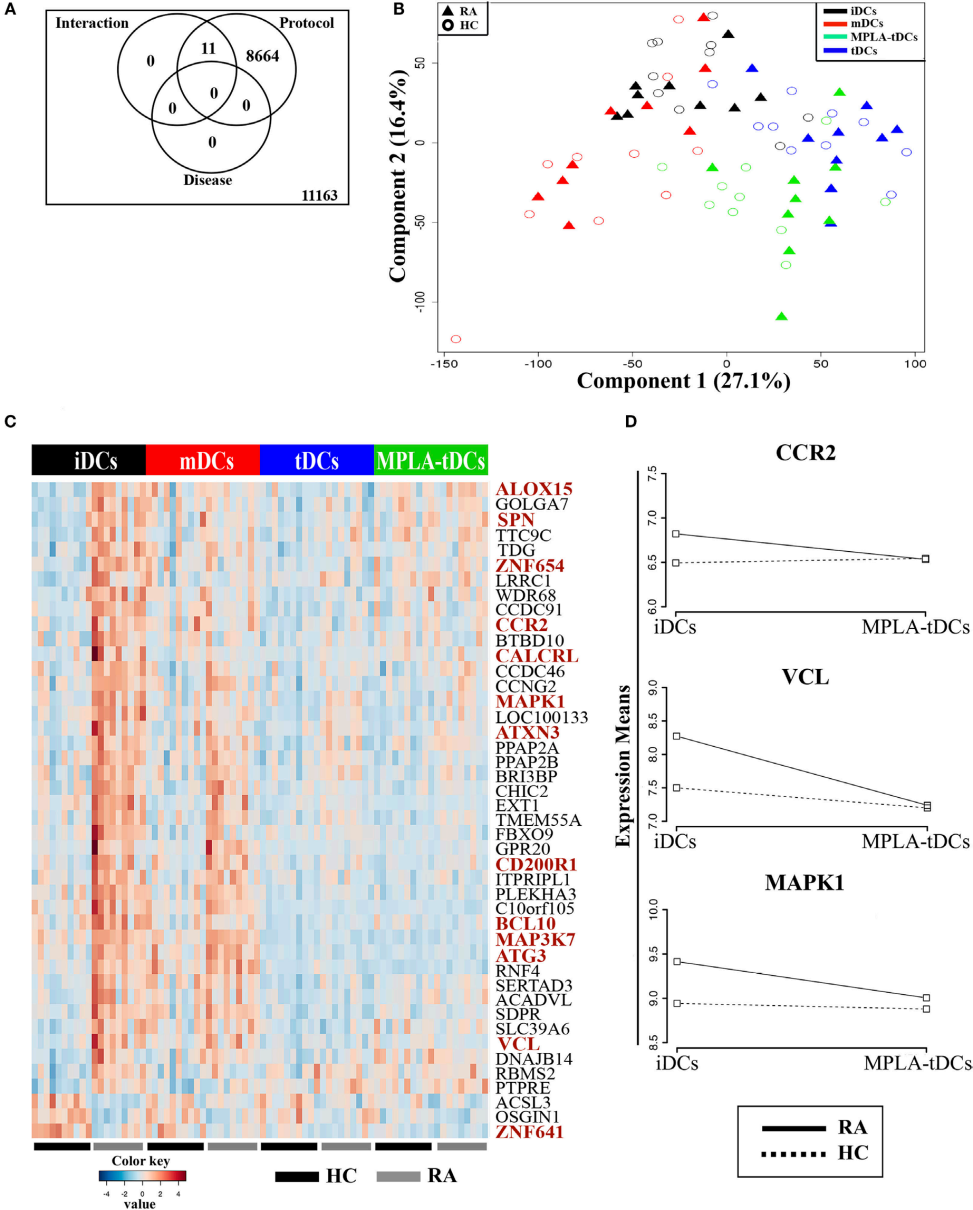
**Conditioning with dexamethasone and MPLA induces similar transcriptional profiles on monocyte-derived DCs from rheumatoid arthritis patients and healthy controls and counterpoints disease-associated effects on gene expression**. **(A)** Venn diagram displaying numbers of differentially expressed (DE) transcripts due to differentiation protocol (iDCs, mDCs, tDCs, or MPLA-tDCs), presence of disease (healthy controls: HC, or rheumartoid arthritis: RA) or interaction of protocol and disease. **(B)** Principal component analysis using the first two components separates samples according to their variance. **(C,D)** Pairwise comparison between moDCs from RA patients and HC were performed for each differentiation protocol separately using *t*-test. **(C)** 44 DE genes found exclusively in iDCs were hierarchically clustered. Genes with known or potential association to RA are highlighted in red color. **(D)** Changes in means of expression of selected RA-associated DE genes in iDCs vs. MPLA-tDCs were shown for HC and RA patients. iDCs, unstimulated immature DCs; tDCs, dexamethasone-modulated TolDCs; mDCs, MPLA-matured DCs; MPLA-tDCs, dexamethasone-modulated MPLA-activated TolDCs.

To confirm that modulation with dexamethasone and MPLA confers tolerogenic features to DCs, regardless of whether they are derived from healthy subjects or RA patients, we analyzed transcriptomes of four different DC preparations, generated from monocytes of healthy donors and RA patients. RNA samples from different DCs preparations were subjected to whole-genome microarray analysis and DE genes between samples were defined by FDR of 0.05 or lower (Figure S1 in Supplementary Material). First, we investigated the effect of different stimuli for DC generation/modulation (“protocol”), health state of the donor (“disease”), or the combination of both (“interaction”), on gene expression (Figure 5). The differentiation protocol (unstimulated: iDCs, dexamethasone: tDCs, MPLA: mDCs, and dexamethasone plus MPLA: MPLA-tDCs) had the main impact on gene expression, affecting 43% of the DC transcriptome (Figure 5A). The “disease” factor alone did not affect global gene expression, while the expression of 11 transcripts was altered by a disease–protocol interaction (Figure 5A; Table S2 in Supplementary Material). This was in agreement with a Principal Component Analysis (PCA), in which the first two dimensions (Component 1, 27.1% variance; Component 2, 16.4% variance) noticeably separated the samples according to the protocol, while no separation between RA and healthy donors was detected (Figure 5B).

To unravel potential effects of RA on gene expression, which might have been missed by the previously applied three-factor model, we performed pairwise comparisons between RA- and healthy donor-derived DCs separately for each protocol. Only when comparing untreated DCs (iDCs) of RA patients and healthy controls (HCs), the analysis revealed 44 DE transcripts (Figure 5C). Differences in the expression of these genes were also present in mDCs, albeit not significant, and completely disappeared in tDCs and MPLA-tDCs (Figure 5C). Among these 44 DE genes, we identified 12 genes with known association to RA (Figure 5C; Table S2 in Supplementary Material) due to their involvement in inflammatory processes, such as NF-kB activation (BCL10, MAP3K7/TAK1, MAPK1/ERK), inflammation (ALOX15, MAPK1/ERK, ATG3, ATXN3, VCL), induction of differentiation and/or activation of Th17 cells (BCL10, CD200R1), as well as chemotaxis and cellular infiltration of synovia (CCR2, CD200R1, SPN/CD43) (36–40). In contrast to HCs, the expression of these RA-associated genes changed markedly in DCs upon modulation with dexamethasone alone or in combination with MPLA (Figures 5C,D). Expression of RA-associated genes, which were upregulated in iDCs of RA patients, declined to levels of HCs in RA-derived MPLA-tDCs. Therefore, modulation with dexamethasone and MPLA removed differences at transcriptional level between moDCs from RA patients and healthy individuals.

## Discussion

As prerequisite for clinical trials using TolDCs for RA therapy, differentiation protocols have to be tested with cells from patients. Here we demonstrate that treatment with dexamethasone and MPLA overcomes disease-associated effects on gene transcription in moDCs and promotes a tolerogenic phenotype with reduced capacity to induce inflammatory responses in antigen-specific T cells.

Dexamethasone, a corticosteroid widely used to treat autoimmune disorders, has been shown to modulate maturation and function of DCs *in vitro* and *in vivo*, validating its use for TolDC generation (41, 42). Additionally, activation through TLR4 is required to endow TolDCs with the ability to process and present exogenous antigen on MHC class II molecules and to acquire the capacity to migrate to secondary lymphoid tissues, the sites of interaction with naïve T cells (43). Functionally, glucocorticoids, such as dexamethasone, suppress pro-inflammatory programs induced by TLR activation, while potentiating TLR-induced anti-inflammatory responses (44, 45). To meet cGMP requirements, we used the non-toxic TLR4 ligand, MPLA, instead of LPS, for DC activation. MPLA exhibits a potent immunostimulatory capacity (46), albeit less than LPS, and has been proven to be an effective and safe adjuvant in animal studies and clinical trials testing next-generation vaccines (43, 47). We previously demonstrated that dexamethasone-modulated and MPLA-activated DCs display a similar tolerogenic phenotype as DCs treated with dexamethasone alone, indicating that MPLA does not affect immunoregulatory properties of dexamethasone-conditioned DCs (20). Stoop and co-workers also demonstrated in an experimental arthritis model that DCs modulated with dexamethasone and vitamin D3, and activated with LPS retained their tolerogenic functions, even in a pro-inflammatory environment *in vivo* (48). According to previous reports describing the properties of TolDCs derived from healthy donors (31, 43), MPLA-tDCs from RA patients showed reduced expression of co-stimulatory molecule CD86, the coactivator CD40, and the maturation marker CD83, as well as high expression of MHC class II, similar to mDCs. Since these cells will encounter a highly pro-inflammatory environment when transferred into RA patients, it is crucial that they are able to retain their regulatory features under these conditions. Exposure of MPLA-tDCs to inflammatory stimuli, such as TNF and SF, which are also present in RA, did not affect their tolerogenic phenotype. Accordingly, their capacity to induce hyporresponsiveness of autologous CD4+ T cells was not impaired upon challenge with SF. The anti-inflammatory cytokine profile of MPLA-tDCs from RA patients points to their immunomodulatory abilities. Correspondingly, RA patient-derived MPLA-tDCs did not promote Th1 and Th17 responses, probably due to reduced IL-12p70 and IL-23 production, and the suppressive effect of other MPLA-tDC-derived molecules, such as IL-10 and TGF-β. This induction of T cell hyporesponsiveness is in accordance with our recent findings, unraveling that MPLA-tDCs from HCs do not promote classical Treg, but rather induce hyporesponsive anergic T cells capable of suppressing Th1 and Th17 responses (49).

Lymph node trafficking is a critical feature of TolDCs when intended to use for cellular therapy in autoimmunity (50). We showed that a significant proportion of MPLA-tDCs from RA patients expressed lymph node homing receptors CCR7 and CXCR4 and migrated *in vitro* in response to CCL19 and CXCL12, albeit less than mDCs. It is conceivable that inoculated MPLA-tDCs might exert their tolerogenic functions not only in lymph nodes but also locally within the inflamed joint, since it has been shown that dexamethasone-treated LPS-activated TolDCs express also CXCR3 and migrate toward its ligand CXCL10/IP10 associated with inflamed tissues (51).

The capacity of TolDCs to present antigen in a MHC class II context is critical for the induction of auto-antigen-specific tolerance (18). The fact that MPLA-tDCs from RA patients express HLA-DR in amounts comparable to mDCs indicates that dexamethasone does not affect antigen presentation and that the poor T cell stimulatory capacity exhibited by MPLA-tDCs in allogeneic and autologous settings might rather be due to their anti-inflammatory properties than to an impairment of antigen presentation. We have recently demonstrated that MPLA-tDCs derived from HLA-DRB1*0101+ RA patients are capable of efficiently presenting collagen peptide hCII(259–273) to the HLA-DR-restricted T cell hybridoma HCII-9.1 (49). Here, we demonstrate that MPLA-tDCs from RA patients are furthermore able to maintain their phenotypic features under inflammatory conditions and to induce hyporesponsiveness of autologous CD4+ T cells with specificity to synovial antigens. This is crucial considering that Th17 and Th1 responses lead to the progression of RA (52).

Monocytes from RA patients are highly activated and inflammatory (28, 29), suggesting that an early transcriptional program in monocytes might favor the development of highly inflammatory DCs *in vivo* and *in vitro*. This might also interfere with the acquirement of regulatory functions when generating TolDCs. Estrada-Capetillo and colleagues reported that moDCs from RA patients release high amounts of IL-6 and IL-23, and exhibit an increased capability to induce Th17 cells, while RA-derived TolDCs failed to induce Treg (30). However, Dr. Hilkens’ group (31) and we have demonstrated that TolDCs generated from monocytes of RA patients possess tolerogenic features comparable to those of healthy individuals. The contradictory results concerning TolDC functionality lead us to further investigate these cells to a transcriptional level in order to identify or discard differences in gene expression between RA and HCs that could interfere with TolDC differentiation and function. Whole-genome expression analysis allowed us to confirm at a transcriptional level that treatment of moDCs with dexamethasone plus MPLA is sufficient to overcome RA-associated effects on gene expression in these cells. Despite individual data scattering, the transcriptional program of MPLA-tDCs remains consistent and allows clear separation from other DCs populations, particularly from untreated (iDCs) and mDCs, which still present disease-related effects on gene expression. This is in accordance with the absence of phenotypic and functional differences between MPLA-tDCs of RA patients and healthy subjects and suggests that MPLA-tDCs are suitable for autologous cellular therapy of RA.

## Conclusion

Combined treatment with dexamethasone and MPLA overcomes RA-associated alterations in gene expression and induces TolDCs with a stable semi-mature phenotype, which migrate toward lymphoid chemokines, secrete regulatory cytokines, and induce hyporesponsiveness of synovial antigens-specific T cells. Thus, MPLA-tDCs are eligible for the development of an autologous tolerance-inducing cellular therapy for the treatment of RA.

## Author Contributions

All authors were involved in drafting the article or revising it critically for important intellectual content, and all authors read and approved the final version of the manuscript. Dr. Aguillón had full access to all of the data in the study and takes responsibility for the integrity of the data and the accuracy of the data analysis. JA, CH, RT, DC, PG-G, and KS participated in study conception and design. LS, ON, and MC were responsible of recruitment and clinical evaluation of RA patients. PG-G, KS, JM, LH, BP, RM, AM, HN, ML, RV, and MM participated in acquisition of data. PG-G, KS, AS-G, AM, HN, RV, JA, CH, RT, and DC participated in analysis and interpretation of data and manuscript writing.

## Conflict of Interest Statement

The authors declare that the research was conducted in the absence of any commercial or financial relationships that could be construed as a potential conflict of interest.

## Abbreviations

ACR, American College of Rheumatology; CCR7, chemokine receptor 7; CFSE, carboxyfluorescein diacetate succinimidyl ester; CXCR4, chemokine receptor 4; CXB, celecoxib; DCs, dendritic cells; ELISA, enzyme linked immune-sorbent assay; FBS, fetal bovine serum; HCQ, hydroxychloroquine; IFN-γ, interferon-γ; IL, interleukin; LFN, leflunomide; LPS, lipopolysaccharide; MEL, meloxicam; MHC, major histocompatibility complex; MPLA, monophosphoryl lipid A; MPLA-tDCs, dexamethasone and monophosphoryl lipid A treated dendritic cells; MPSL, methylprednisolone; MTX, methotrexate; NSAID, non-steroidal anti-inflammatory drugs; PDN, prednisone; RA, rheumatoid arthritis; rhGM-CSF, recombinant human granulocyte-macrophage colony stimulating factor; SF, synovial fluid; TGF-β, transforming growth factor beta; TLR2, toll-like receptor 2; TolDCs, tolerogenic dendritic cells; Treg, regulatory T cells.

## References

[1] Wang W Shao S Jiao Z Guo M Xu H Wang S. The Th17/Treg imbalance and cytokine environment in peripheral blood of patients with rheumatoid arthritis. Rheumatol Int (2012) 32(4):887–93. 10.1007/s00296-010-1710-0 21221592

[2] Schinnerling K Soto L Garcia-Gonzalez P Catalan D Aguillon JC. Skewing dendritic cell differentiation towards a tolerogenic state for recovery of tolerance in rheumatoid arthritis. Autoimmun Rev (2015) 14(6):517–27. 10.1016/j.autrev.2015.01.014 25633325

[3] Tsark EC Wang W Teng YC Arkfeld D Dodge GR Kovats S. Differential MHC class II-mediated presentation of rheumatoid arthritis autoantigens by human dendritic cells and macrophages. J Immunol (2002) 169(11):6625–33. 10.4049/jimmunol.169.11.6625 12444176

[4] Lutzky V Hannawi S Thomas R Cells of the synovium in rheumatoid arthritis. Dendritic cells. Arthritis Res Ther (2007) 9(4):219 10.1186/ar2200 17850683 PMC2206384

[5] Canete JD Pablos JL Biologic therapy in rheumatoid arthritis. Curr Top Med Chem (2013) 13(6):752–9. 10.2174/15680266113139990093 23574524

[6] Lutz MB. Therapeutic potential of semi-mature dendritic cells for tolerance induction. Front Immunol (2012) 3:123. 10.3389/fimmu.2012.00123 22629255 PMC3355325

[7] Thomas R. Dendritic cells as targets or therapeutics in rheumatic autoimmune disease. Curr Opin Rheumatol (2014) 26(2):211–8. 10.1097/bor.0000000000000032 24389864

[8] Torres-Aguilar H Aguilar-Ruiz SR Gonzalez-Perez G Munguia R Bajana S Meraz-Rios MA Tolerogenic dendritic cells generated with different immunosuppressive cytokines induce antigen-specific anergy and regulatory properties in memory CD4+ T cells. J Immunol (2010) 184(4):1765–75. 10.4049/jimmunol.0902133 20083662

[9] Luckey U Maurer M Schmidt T Lorenz N Seebach B Metz M T cell killing by tolerogenic dendritic cells protects mice from allergy. J Clin Invest (2011) 121(10):3860–71. 10.1172/jci45963 21881208 PMC3195460

[10] Gregori S Tomasoni D Pacciani V Scirpoli M Battaglia M Magnani C Differentiation of type 1 T regulatory cells (Tr1) by tolerogenic DC-10 requires the IL-10–dependent ILT4/HLA-G pathway. Blood (2010) 116(6):935–44. 10.1182/blood-2009-07-234872 20448110

[11] Huang H Dawicki W Zhang X Town J Gordon JR. Tolerogenic dendritic cells induce CD4+CD25hiFoxp3+ regulatory T cell differentiation from CD4+CD25-/loFoxp3- effector T cells. J Immunol (2010) 185(9):5003–10. 10.4049/jimmunol.0903446 20870943

[12] Azzaoui I Yahia SA Chang Y Vorng H Morales O Fan Y CCL18 differentiates dendritic cells in tolerogenic cells able to prime regulatory T cells in healthy subjects. Blood (2011) 118(13):3549–58. 10.1182/blood-2011-02-338780 21803856

[13] Fogel-Petrovic M Long JA Misso NL Foster PS Bhoola KD Thompson PJ. Physiological concentrations of transforming growth factor beta1 selectively inhibit human dendritic cell function. Int Immunopharmacol (2007) 7(14):1924–33. 10.1016/j.intimp.2007.07.003 18039529

[14] Escobar A Aguirre A Guzman MA Gonzalez R Catalan D Acuna-Castillo C Tolerogenic dendritic cells derived from donors with natural rubber latex allergy modulate allergen-specific T-cell responses and IgE production. PLoS One (2014) 9(1):e85930. 10.1371/journal.pone.0085930 24465795 PMC3899084

[15] Benham H Nel HJ Law SC Mehdi AM Street S Ramnoruth N Citrullinated peptide dendritic cell immunotherapy in HLA risk genotype-positive rheumatoid arthritis patients. Sci Transl Med (2015) 7(290):290ra287. 10.1126/scitranslmed.aaa9301 26041704

[16] Matsue H Yang C Matsue K Edelbaum D Mummert M Takashima A. Contrasting impacts of immunosuppressive agents (rapamycin, FK506, cyclosporin A, and dexamethasone) on bidirectional dendritic cell-T cell interaction during antigen presentation. J Immunol (2002) 169(7):3555–64. 10.4049/jimmunol.169.7.3555 12244145

[17] Buckland M Jago C Fazekesova H George A Lechler R Lombardi G. Aspirin modified dendritic cells are potent inducers of allo-specific regulatory T-cells. Int Immunopharmacol (2006) 6(13–14):1895–901. 10.1016/j.intimp.2006.07.008 17219690

[18] Anderson AE Sayers BL Haniffa MA Swan DJ Diboll J Wang XN Differential regulation of naive and memory CD4+ T cells by alternatively activated dendritic cells. J Leukoc Biol (2008) 84(1):124–33. 10.1189/jlb.1107744 18430785 PMC2504714

[19] Salazar L Aravena O Abello P Escobar A Contreras-Levicoy J Rojas-Colonelli N Modulation of established murine collagen-induced arthritis by a single inoculation of short-term lipopolysaccharide-stimulated dendritic cells. Ann Rheum Dis (2008) 67(9):1235–41. 10.1136/ard.2007.072199 18056756

[20] Garcia-Gonzalez P Morales R Hoyos L Maggi J Campos J Pesce B A short protocol using dexamethasone and monophosphoryl lipid A generates tolerogenic dendritic cells that display a potent migratory capacity to lymphoid chemokines. J Transl Med (2013) 11:128. 10.1186/1479-5876-11-128 23706017 PMC3674980

[21] Steinbrink K Graulich E Kubsch S Knop J Enk AH. CD4(+) and CD8(+) anergic T cells induced by interleukin-10-treated human dendritic cells display antigen-specific suppressor activity. Blood (2002) 99(7):2468–76. 10.1182/blood.V99.7.2468 11895781

[22] Naranjo-Gomez M Raich-Regue D Onate C Grau-Lopez L Ramo-Tello C Pujol-Borrell R Comparative study of clinical grade human tolerogenic dendritic cells. J Transl Med (2011) 9:89. 10.1186/1479-5876-9-89 21658226 PMC3141500

[23] Martin E Capini C Duggan E Lutzky VP Stumbles P Pettit AR Antigen-specific suppression of established arthritis in mice by dendritic cells deficient in NF-kappaB. Arthritis Rheum (2007) 56(7):2255–66. 10.1002/art.22655 17599748

[24] Garate D Rojas-Colonelli N Pena C Salazar L Abello P Pesce B Blocking of p38 and transforming growth factor beta receptor pathways impairs the ability of tolerogenic dendritic cells to suppress murine arthritis. Arthritis Rheum (2013) 65(1):120–9. 10.1002/art.37702 22972370

[25] Giannoukakis N Phillips B Finegold D Harnaha J Trucco M. Phase I (safety) study of autologous tolerogenic dendritic cells in type 1 diabetic patients. Diabetes Care (2011) 34(9):2026–32. 10.2337/dc11-0472 21680720 PMC3161299

[26] Bell GM Anderson AE Diboll J Reece R Eltherington O Harry RA Autologous tolerogenic dendritic cells for rheumatoid and inflammatory arthritis. Ann Rheum Dis (2016). 10.1136/annrheumdis-2015-208456 27117700 PMC5264217

[27] Abbas AK Le K Pimmett VL Bell DA Cairns E Dekoter RP Negative regulation of the peptidylarginine deiminase type IV promoter by NF-kappaB in human myeloid cells. Gene (2014) 533(1):123–31. 10.1016/j.gene.2013.09.108 24140127

[28] Davignon J-LL Hayder M Baron M Boyer J-FF Constantin A Apparailly F Targeting monocytes/macrophages in the treatment of rheumatoid arthritis. Rheumatology (Oxford) (2013) 52(4):590–8. 10.1093/rheumatology/kes304 23204551

[29] Ruscitti P Cipriani P Di Benedetto P Liakouli V Berardicurti O Carubbi F Monocytes from patients with rheumatoid arthritis and type 2 diabetes mellitus display an increased production of interleukin (IL)-1beta via the nucleotide-binding domain and leucine-rich repeat containing family pyrin 3(NLRP3)-inflammasome activation: a possible implication for therapeutic decision in these patients. Clin Exp Immunol (2015) 182(1):35–44. 10.1111/cei.12667 26095630 PMC4578506

[30] Estrada-Capetillo L Hernandez-Castro B Monsivais-Urenda A Alvarez-Quiroga C Layseca-Espinosa E Abud-Mendoza C Induction of Th17 lymphocytes and Treg cells by monocyte-derived dendritic cells in patients with rheumatoid arthritis and systemic lupus erythematosus. Clin Dev Immunol (2013) 2013:584303. 10.1155/2013/584303 24288552 PMC3830818

[31] Harry RA Anderson AE Isaacs JD Hilkens CM. Generation and characterisation of therapeutic tolerogenic dendritic cells for rheumatoid arthritis. Ann Rheum Dis (2010) 69(11):2042–50. 10.1136/ard.2009.126383 20551157 PMC3002758

[32] Lord P Spiering R Aguillon JC Anderson AE Appel S Benitez-Ribas D Minimum information about tolerogenic antigen-presenting cells (MITAP): a first step towards reproducibility and standardisation of cellular therapies. PeerJ (2016) 4:e2300. 10.7717/peerj.2300 27635311 PMC5012269

[33] Le Cao K-A González I Dejean S mixOmics: Omics Data Integration Project. R package version 5.0–3. (2014). Available at: https://CRAN.R-project.org/package=mixOmics

[34] Team RC R: A Language and Environment for Statistical Computing. Vienna, Austria: R Foundation for Statistical Computing (2014).

[35] Wu H Kerr MK Cui X Churchill GA MAANOVA: a software package for the analysis of spotted cDNA microarray experiments. In: Parmigiani G Garrett ES Irizarry RA Zeger SL, editors. The Analysis of Gene Expression Data: Methods and Software. New York, NY: Springer (2003). p. 313–41.

[36] Lu MC Lai NS Yin WY Yu HC Huang HB Tung CH Anti-citrullinated protein antibodies activated ERK1/2 and JNK mitogen-activated protein kinases via binding to surface-expressed citrullinated GRP78 on mononuclear cells. J Clin Immunol (2013) 33(3):558–66. 10.1007/s10875-012-9841-6 23188524

[37] Shinohara H Behar M Inoue K Hiroshima M Yasuda T Nagashima T Positive feedback within a kinase signaling complex functions as a switch mechanism for NF-kappaB activation. Science (2014) 344(6185):760–4. 10.1126/science.1250020 24833394

[38] Wang H Zhao J Zhang H Huang Y Wang S Tu Q CARD11 blockade suppresses murine collagen-induced arthritis via inhibiting CARD11/Bcl10 assembly and T helper type 17 response. Clin Exp Immunol (2014) 176(2):238–45. 10.1111/cei.12275 24443940 PMC3992036

[39] Ren Y Yang B Yin Y Leng X Jiang Y Zhang L Aberrant CD200/CD200R1 expression and its potential role in Th17 cell differentiation, chemotaxis and osteoclastogenesis in rheumatoid arthritis. Rheumatology (Oxford) (2015) 54(4):712–21. 10.1093/rheumatology/keu362 25261692

[40] van Heemst J Jansen DT Polydorides S Moustakas AK Bax M Feitsma AL Crossreactivity to vinculin and microbes provides a molecular basis for HLA-based protection against rheumatoid arthritis. Nat Commun (2015) 6:6681. 10.1038/ncomms7681 25942574

[41] Woltman AM van der Kooij SW de Fijter JW van Kooten C. Maturation-resistant dendritic cells induce hyporesponsiveness in alloreactive CD45RA+ and CD45RO+ T-cell populations. Am J Transplant (2006) 6(11):2580–91. 10.1111/j.1600-6143.2006.01520.x 16952295

[42] van Duivenvoorde LM Han WG Bakker AM Louis-Plence P Charbonnier LM Apparailly F Immunomodulatory dendritic cells inhibit Th1 responses and arthritis via different mechanisms. J Immunol (2007) 179(3):1506–15. 10.4049/jimmunol.179.3.1506 17641016

[43] Anderson AE Swan DJ Sayers BL Harry RA Patterson AM von Delwig A LPS activation is required for migratory activity and antigen presentation by tolerogenic dendritic cells. J Leukoc Biol (2009) 85(2):243–50. 10.1189/jlb.0608374 18971286 PMC2700018

[44] Ogawa S Lozach J Benner C Pascual G Tangirala RK Westin S Molecular determinants of crosstalk between nuclear receptors and toll-like receptors. Cell (2005) 122(5):707–21. 10.1016/j.cell.2005.06.029 16143103 PMC1430687

[45] Søndergaard JN Poghosyan S Hontelez S Louche P Looman MW Ansems M DC-SCRIPT regulates IL-10 production in human dendritic cells by modulating NF-κBp65 activation. J Immunol (2015) 195(4):1498–505. 10.4049/jimmunol.1402924 26170389

[46] Ismaili J Rennesson J Aksoy E Vekemans J Vincart B Amraoui Z Monophosphoryl lipid A activates both human dendritic cells and T cells. J Immunol (2002) 168(2):926–32. 10.4049/jimmunol.168.2.926 11777991

[47] Casella CR Mitchell TC. Putting endotoxin to work for us: monophosphoryl lipid A as a safe and effective vaccine adjuvant. Cell Mol Life Sci (2008) 65(20):3231–40. 10.1007/s00018-008-8228-6 18668203 PMC2647720

[48] Stoop JN Harry RA von Delwig A Isaacs JD Robinson JH Hilkens CM. Therapeutic effect of tolerogenic dendritic cells in established collagen-induced arthritis is associated with a reduction in Th17 responses. Arthritis Rheum (2010) 62(12):3656–65. 10.1002/art.27756 20862679

[49] Maggi J Schinnerling K Pesce B Hilkens C Catalán D Aguillón J Dexamethasone and monophosphoryl lipid A-modulated dendritic cells promote antigen-specific tolerogenic properties on naïve and memory CD4+ T cells. Front Immunol (2016) 7:359 10.3389/fimmu.2016.00359 27698654 PMC5027201

[50] Ochando JC Yopp AC Yang Y Garin A Li Y Boros P Lymph node occupancy is required for the peripheral development of alloantigen-specific Foxp3+ regulatory T cells. J Immunol (2005) 174(11):6993–7005. 10.4049/jimmunol.174.11.6993 15905542

[51] Unger WW Laban S Kleijwegt FS van der Slik AR Roep BO. Induction of Treg by monocyte-derived DC modulated by vitamin D3 or dexamethasone: differential role for PD-L1. Eur J Immunol (2009) 39(11):3147–59. 10.1002/eji.200839103 19688742

[52] Santiago-Schwarz F Anand P Liu S Carsons SE. Dendritic cells (DCs) in rheumatoid arthritis (RA): progenitor cells and soluble factors contained in RA synovial fluid yield a subset of myeloid DCs that preferentially activate Th1 inflammatory-type responses. J Immunol (2001) 167(3):1758–68. 10.4049/jimmunol.167.3.1758 11466401

