# Supplementary material for: Treatment with Dexamethasone and Monophosphoryl Lipid A Removes Disease-Associated Transcriptional Signatures in Monocyte-Derived Dendritic Cells from Rheumatoid Arthritis Patients and Confers Tolerogenic Features
Source: Front Immunol. 2016 Oct 25;7:458. doi: 10.3389/fimmu.2016.00458 (PMC5078319; doi:10.3389/fimmu.2016.00458)
Supplement: Supplementary file 3 [file Data_Sheet_1.docx]

Supplementary Material

**“Treatment with dexamethasone and monophosphoryl lipid A removes disease-associated transcriptional signatures in monocyte-derived dendritic cells from rheumatoid arthritis patients and confers tolerogenic features”**

**Paulina García-González^1,2^, Katina Schinnerling^1,2^, Alejandro Sepúlveda-Gutiérrez^3^, Jaxaira Maggi^1,2^, Lorena Hoyos^1,2^, Rodrigo Morales^1,2^, Ahmed M. Mehdi^4^, Hendrik J. Nel^4^, Lilian Soto^1,5^, Bárbara Pesce^1,2^, María Carmen Molina^1^, Miguel Cuchacovich^6^, Milton L. Larrondo^7^, Óscar Neira^7^, Diego Catalán^1,2^, Catharien M. Hilkens^8^, Ranjeny Thomas^4^, Ricardo A. Verdugo^3^, Juan C. Aguillón^1,2*^**

*** Correspondence:** Dr. Juan C. Aguillón: [jaguillo@med.uchile.cl](mailto:jaguillo@med.uchile.cl)

**Supplementary Figure 1**

##
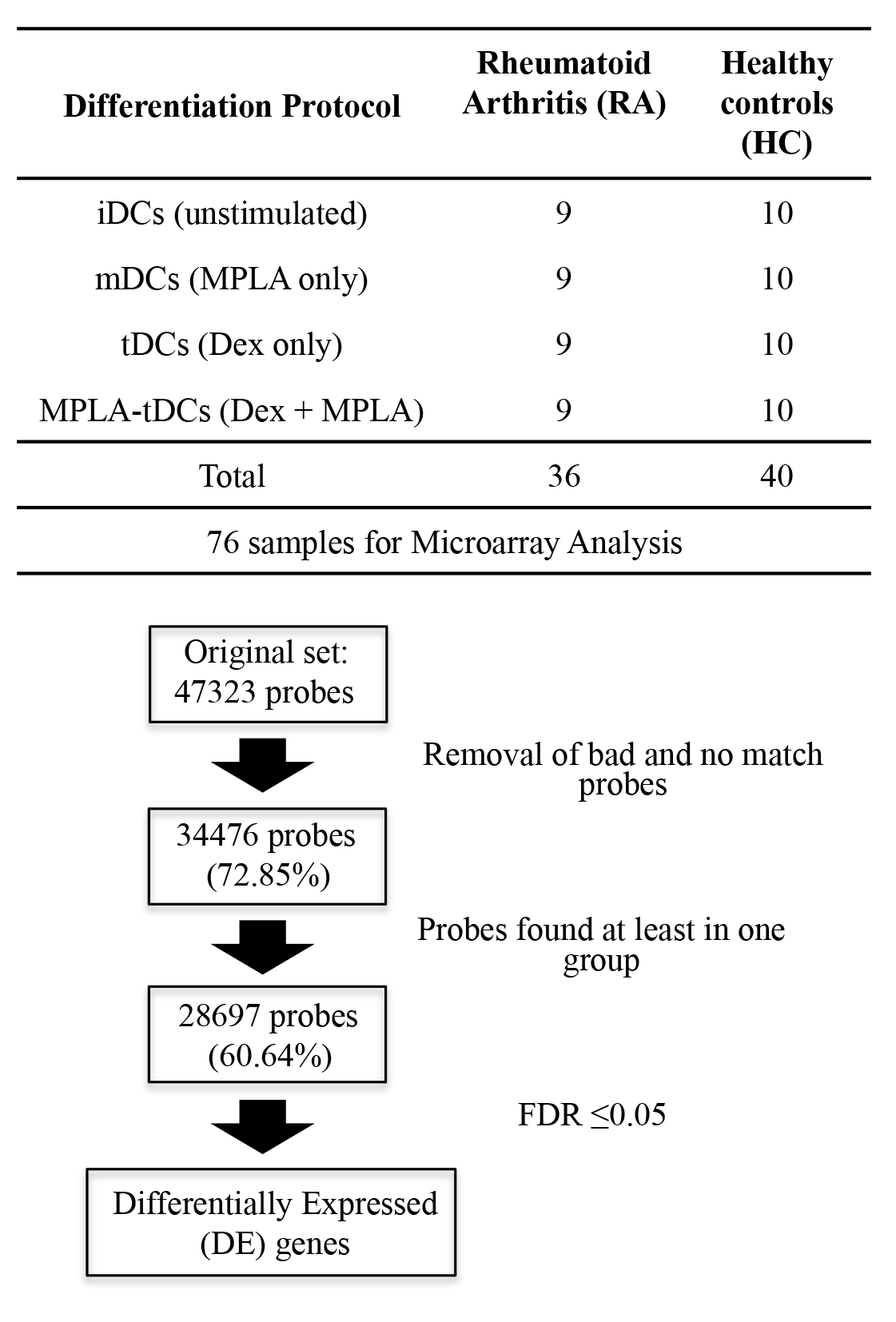


**Supplementary Figure 1. Microarray analysis settings.** Total RNA was isolated from monocyte-derived DCs (moDCs) of 9 rheumatoid arthritis (RA) patients and 10 healthy controls (HC) generated under 4 different experimental conditions (Dex-treated DCs, MPLA-matured DCs, Dex-treated/MPLA-matured DCs and immature/untreated DCs). moDCs were phenotypically and functionally characterized prior to RNA extraction. A total of 76 samples, corresponding to DCs generated under 4 experimental conditions were considered for analysis and classified by both differentiation protocol and patient condition (RA or HC). Microarray data was log-transformed and normalized using the preprocess Core package v1.28.0 from Bioconductor. The R software v3.1.3 (R Core Team, 2015) was used for data analysis. The dataset was normalized using quantiles.normalization function (preprocessCore package v1.28.0) in order to match intensity scale between sample data. Dataset filtering included probe quality, detection and gene expression differences among treatments. Initial dataset filtering included removal of probes with bad and no match annotations from the IlluminaHumanv4 database (IlluminaHumanv4fullReannotation, R software). Only probes with a detection p-value equivalent or higher than 0.95 in at least one experimental group were considered. Principal Component analysis was performed using the pca function from R software, mixOmics package v5.0- 3 (Le Cao et al., 2014; Team, 2014), to assess differences in differentiation protocols and the presence of samples showing outlier expression values that could influence downstream analyses. Analysis of differential expression (DE) of genes in modulated DCs compared to untreated iDCs was performed with three different ANOVA models by means of the fitmaanova function with the Maanova package v1.36.0 using 1000 permutations (Wu et al., 2003). The first model considered the disease factor presence and differentiation protocols as isolated factors, in order to assess differences between sample sets that were independent of potential interactions. The second model added the interaction between disease (RA) and differentiation protocol, and the third one was fitted considering disease and differentiation protocol as a single factor. The “donor” factor was modeled as random in all formulas. These three models were used for five DE tests performed using the matest function (maanova package v1.36.0). F test was applied to the first three analyses to assess the effect of disease, differentiation protocol and interaction between these factors on gene expression. For the other two analyses, pairwise comparison of samples was performed using t-test in order to find DE genes between RA and HC specific for each experimental condition. F test statistics and their corresponding p-values were extracted and corrected using the adjPval function from the maanova package to obtain false discovery rate (FDR) values using the adaptive method. Cutoff values of FDR 0.05 or lower and a fold change of 1.5 were used to define DE genes. K-means clustering of DE genes between MPLA-tDCs and iDCs was performed using the cluster package.

Le Cao K-A, González I, Dejean S. mixOmics: Omics Data Integration Project. R package version 5.0-3. (2014).https://CRAN.R-project.org/package=mixOmics

Team, R.C. (2014). "R: A Language and Environment for Statistical Computing". (Vienna, Austria: R Foundation for Statistical Computing).

Wu, H., Kerr, M.K., Cui, X., and Churchill, G.A. (2003). "MAANOVA: A Software Package for the Analysis of Spotted cDNA Microarray Experiments," in *The Analysis of Gene Expression Data: Methods and Software,* eds. G. Parmigiani, E.S. Garrett, R.A. Irizarry & S.L. Zeger. (New York, NY: Springer New York), 313-341.
